# The Intrinsic Radius as a Key Parameter in the Generalized Born Model to Adjust Protein-Protein Electrostatic Interaction

**DOI:** 10.3390/ijms24054700

**Published:** 2023-02-28

**Authors:** Dan Parkin, Mitsunori Takano

**Affiliations:** 1 Research Institute for Science and Engineering, Waseda University, Okubo 3-4-1, Sinjuku-ku, Tokyo 169-8555, Japan; 2 Department of Pure and Applied Physics, Waseda University, Okubo 3-4-1, Sinjuku-ku, Tokyo 169-8555, Japan

**Keywords:** Coulomb interaction, molecular dynamics simulation, GB model, desolvation energy, charge complementarity, protein–protein interaction

## Abstract

The generalized Born (GB) model is an extension of the continuum dielectric theory of Born solvation energy and is a powerful method for accelerating the molecular dynamic (MD) simulations of charged biological molecules in water. While the effective dielectric constant of water that varies as a function of the separation distance between solute molecules is incorporated into the GB model, adjustment of the parameters is indispensable for accurate calculation of the Coulomb (electrostatic) energy. One of the key parameters is the lower limit of the spatial integral of the energy density of the electric field around a charged atom, known as the intrinsic radius ρ. Although ad hoc adjustment of ρ has been conducted to improve the Coulombic (ionic) bond stability, the physical mechanism by which ρ affects the Coulomb energy remains unclear. Via energetic analysis of three differently sized systems, here, we clarify that the Coulomb bond stability increases with increasing ρ and that the increased stability is caused by the interaction energy term, not by the self-energy (desolvation energy) term, as was supposed previously. Our results suggest that the use of larger values for the intrinsic radii of hydrogen and oxygen atoms, together with the use of a relatively small value for the spatial integration cutoff in the GB model, can better reproduce the Coulombic attraction between protein molecules.

## 1. Introduction

The Coulomb interaction is important for the assembly of biomolecules and their fast association in aqueous solution [1,2]. The stability of a Coulomb bond (i.e., an ionic or hydrogen bond) in water, however, is marginal compared to that in a vacuum, which is usually understood by the large dielectric constant of water. For the Coulomb interaction between two oppositely charged molecules that have their own water-excluded volumes, the effective dielectric constant is lowered as the two molecules approach each other because of the presence of the water-excluded (and hence, low-dielectric) regions. The lowering of the effective dielectric constant causes augmentation of the attractive Coulomb *interaction* energy of the two molecules and, simultaneously, causes the increase in the Coulomb *self*-energy, which is always positive (in other words, the reduction in the Born solvation energy [3], which is always negative). Thus, the favorable change in the Coulomb interaction energy upon the Coulomb bond formation is counterbalanced by the unfavorable change in the Coulomb self-energy, which makes the stability of the Coulomb bond in water marginal.

The lowering of the effective dielectric constant and the counterbalance between the Coulomb interaction and the self-energies, as mentioned above, are taken into consideration in the generalized Born (GB) solvation model [4] that has been applied to the molecular dynamics (MD) simulation of biomolecular systems in water [5,6,7,8,9,10,11,12,13,14,15,16,17,18,19,20,21,22,23]. The obvious benefit of using the GB model is the speed-up of the MD simulation due to replacing a large number of the solvent degrees of freedom with a continuum dielectric. On the other hand, the adjustment of the parameters employed in the GB model is indispensable to calculating the Coulomb energies accurately [5,6,7,8,9,10,11,12,13,14,15,16,17,18,19,20,21,22,23]. Among the key parameters of the GB model, we previously studied the effect of the upper limit (cutoff distance), denoted by $r_{\max}$, for the spatial integral of the energy density of the electric field around a charged atom [24]. The spatial integral of the energy density of the electric field is used to obtain the effective Born radius of the atom (the most important quantity in the GB model that measures the effective dielectric constant around the atom). We found that $r_{\max}$ affects the electrostatic interaction between charged molecules; the attractive Coulomb interaction between oppositely charged molecules becomes repulsive when a large $r_{\max}$ value is used, which was shown to be caused by the overestimation of the unfavorable Coulomb self-energy (or desolvation penalty) upon binding so that the use of a relatively small $r_{\max}$ value was recommended [24].

As another important parameter of the GB model, the lower limit of the above-mentioned integral, was recognized early [4]. The lower limit of the integral defines the dielectric boundary of the solute region and the solvent region, and the van der Waals radius of the atom (called the Bondi radius [25]) is basically used for this lower limit, which is referred to as the GB atomic radius or the intrinsic radius, denoted by ρ [11,12]. The parameter ρ has been used for the ad hoc adjustment of the stability of the Coulomb bond [11,12,16,19,20,23,26,27]. For example, Tsui and Case augmented the stability of hydrogen bonds between DNA strands by increasing the ρ of the hydrogen atom [11,12]. Im and coworkers reduced the stability of the backbone hydrogen bonds of helix-forming peptides by reducing the ρ of the nitrogen atom [19]. Although it is commonly observed that increasing the ρ leads to the enhancement of the Coulombic (i.e, ionic or hydrogen) bond stability, the underlying physical mechanism has been only speculated from the viewpoint of the reduced desolvation penalty.

In the present study, which forms a counterpart to the previous study [24], we systematically analyze the dependence of the Coulomb *interaction* energy and the Coulomb *self*-energy on ρ and show that the enhancement of the Coulomb bond stability with increasing ρ is caused by the interaction energy term, not by the self-energy (desolvation energy) term, as was previously supposed. We also show that ρ (the lower limit of the spatial integration) affects the Coulomb interaction at short distances, whereas $r_{\max}$ (the upper limit) affects it at long distances, thereby enabling us to use these two parameters in combination. Indeed, we show that larger intrinsic radii, in combination with a relatively small cutoff distance for the spatial integral, can better reproduce the attractive interaction between oppositely charged protein molecules.

## 2. Results and Discussion

We analyzed the effect of the intrinsic radii, ρ, on the Coulomb *self*-energy (i.e., the desolvation energy defined by Equation (13)) and the Coulomb *interaction* energy (Equation (15)) upon the Coulomb bond (ionic bond) formation. We employed three systems for the energy analysis: (i) a two-atom system (H–O system), (ii) a two amino acid system (Arg–Glu system), and (iii) a two-protein system (barstar–barnase system).

### 2.1. Two-Atom System (H–O System)

**Figure 1:**
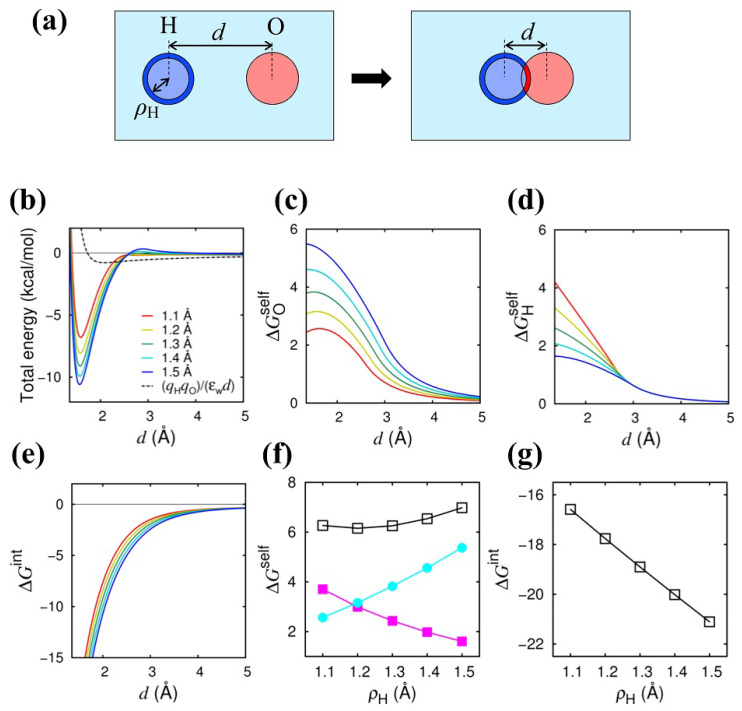
Effect Author request: Please enlarge the figure size for visibility (we changed the ration from 0.8 to 0.95 of $\rho_{H}$ on Coulomb energies in H–O system. (**a**) Illustration of the Coulomb bond formation. Dark-colored regions indicate the regions that are subjected to the integral in Equation (11) and are affected by the increase in $\rho_{H}$. (**b**) Total energy [i.e., the sum of the Coulomb and van der Waals (Lennard–Jones) energies] is shown as a function of the separation distance *d* between the two atoms at different $\rho_{H}$ values (1.1 to 1.5 Å). The dotted line represents the total energy where Equation (3) is used for the Coulomb energy ($\varepsilon_{w}=78.5$). (**c**) Desolvation $\Delta G_{O}^{\mathrm{self}}$, (**d**) desolvation energy $\Delta G_{H}^{\mathrm{self}}$, and (**e**) interaction energy $G^{\mathrm{int}}$ are shown as a function of *d*. Line colors correspond to those in (**b**). (**f**) Desolvation energies [$\Delta G_{O}^{\mathrm{self}}$ (cyan), $\Delta G_{H}^{\mathrm{self}}$ (magenta), and $\Delta G_{O}^{\mathrm{self}}+\Delta G_{H}^{\mathrm{self}}$ (open square)] at d=1.57 Å are shown as a function of $\rho_{H}$. (**g**) Interaction energy $G^{\mathrm{int}}$ at d=1.57 Å is shown as a function of $\rho_{H}$. Energy unit is kcal/mol.

We first show the results of the energetic analysis of the Coulomb bond formation in the two-atom (H–O) system in water, as shown in Figure 1a. The total energy (the sum of the Coulomb and van der Waals energies) is shown in Figure 1b as a function of *d* (the separation distance between the hydrogen and oxygen atoms). We can see that the energy minimum appears at d≈1.6 Å and is lowered as the intrinsic radius of the hydrogen atom, $\rho_{H}$, is increased from 1.1 Å (red) to 1.5 Å (blue), indicating that the Coulomb bond is more stabilized with increasing $\rho_{H}$. This increase in the Coulomb bond stability with increasing $\rho_{H}$ is in accord with what has been observed in the previous studies [11,12,16,23,27]. The effect of $\rho_{H}$ on the total energy is limited to the short distance range (d≲3 Å).

In Figure 1c,d, we show how the Coulomb *self*-energy (i.e., the desolvation energy) and the Coulomb *interaction* energy are affected by $\rho_{H}$. The energy profile of $\Delta G_{O}^{\mathrm{self}}$ [Figure 1c] indicates that the self-energy of the oxygen is increased as *d* is decreased, indicating that the unfavorable desolvation energy, called the “desolvation penalty”, arises upon the Coulomb bond formation. The desolvation penalty is seen in the total energy [Figure 1b] as an energy barrier at d∼3 Å, and the energy barrier is increased as $\rho_{H}$ is increased. The increase in $\Delta G_{O}^{\mathrm{self}}$ with increasing $\rho_{H}$ is understood by inspecting the spatial integral in Equation (11), that is, the *I*-value of the oxygen atom increased with increasing $\rho_{H}$; see the dark blue region in Figure 1a that indicates the increased volume to be integrated in Equation (11). The increase in the *I*-value reflects the lowering of the effective dielectric constant around the oxygen (red). Figure 1d shows that the desolvation penalty upon the Coulomb bond formation arises in $\Delta G_{H}^{\mathrm{self}}$ as well. However, the $\rho_{H}$ dependence of $\Delta G_{H}^{\mathrm{self}}$ is opposite to that of $\Delta G_{O}^{\mathrm{self}}$; it decreases with increasing $\rho_{H}$. This opposite behavior can also be understood by inspecting the spatial integral in Equation (11), that is, the *I*-value of the hydrogen atom decreases with increasing $\rho_{H}$; see the dark red region in the right part of Figure 1a that indicates the reduced volume to be integrated. As displayed in Figure 1f, the increase in $\Delta G_{O}^{\mathrm{self}}$ with increasing $\rho_{H}$ is largely compensated by the decrease in $\Delta G_{H}^{\mathrm{self}}$, so the desolvation energy in total remains almost unchanged (with a slight increase at a larger $\rho_{H}$).

On the other hand, the Coulomb *interaction* energy, $G^{\mathrm{int}}$, as a function of *d* [Figure 1e] decreases as *d* is decreased, stabilizing the Coulomb bond, and the amount of the decrease (stabilization) is enhanced with increasing $\rho_{H}$, which is clearly seen in Figure 1g, in which $G^{\mathrm{int}}$ at the total energy minimum (d=1.57 Å) is shown as a function of $\rho_{H}$. Therefore, we can tell that it is the Coulomb interaction energy $G^{\mathrm{int}}$, not the self (desolvation) energy, as was previously supposed, that contributes to the enhancement of the Coulomb bond stability at a larger $\rho_{H}$. The enhancement of the attractive interaction at a larger $\rho_{H}$ is due to the increased water-excluded (and hence, low-dielectric) region encompassing the two interacting charges.

### 2.2. Two Amino Acid System (Arg–Glu System)

**Figure 2:**
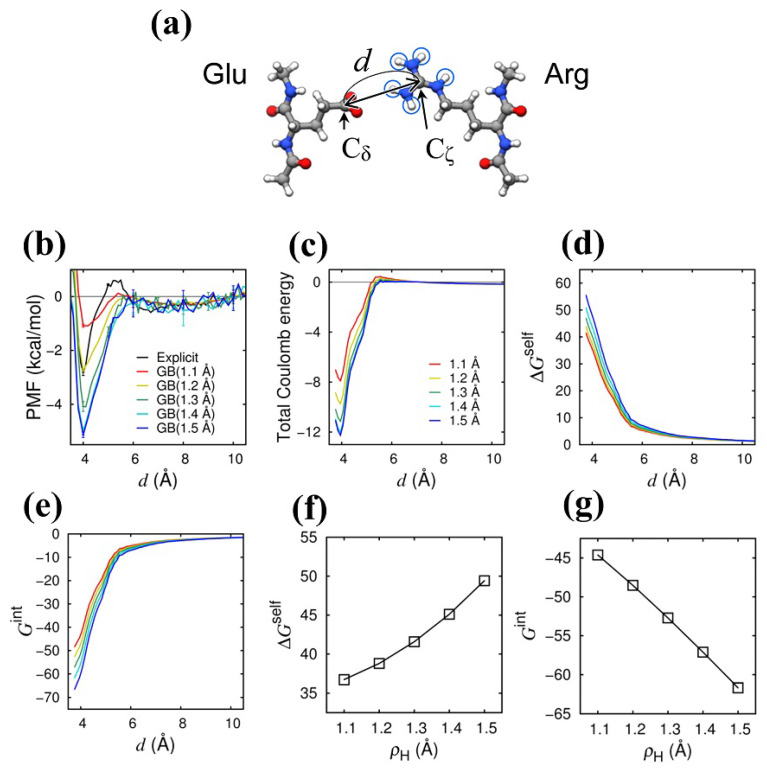
Author request: Please enlarge the figure size for visibility (we changed the ration from 0.8 to 0.95 Effect of $\rho_{H}$ on Coulomb energies in Arg–Glu system. (**a**) Illustration of the system. Encircled hydrogen atoms in the guanine group of Arg are the atoms, the intrinsic radii of which ($\rho_{H}$) were uniformly and systematically changed from 1.1 Å to 1.5 Å. (**b**) Potential of mean force (PMF) is shown as a function of *d* [see (**a**)] for the explicit water model (black) and for the GB solvation model at different $\rho_{H}$ values (1.1 to 1.5 Å). (**c**) Total Coulomb energy ($\Delta G^{\mathrm{self}}+G^{\mathrm{int}}$), (**d**) total desolvation energy ($\Delta G^{\mathrm{self}}$), and (**e**) total Coulomb interaction energy ($G^{\mathrm{int}}$) are shown as a function of *d* at different $\rho_{H}$ values [line colors correspond to those in (**c**)]. (**f**) Total desolvation energy ($\Delta G^{\mathrm{self}}$) and (**g**) total interaction energy ($G^{\mathrm{int}}$) averaged in the range of 3.9≤d≤4.0 Å are shown as a function of $\rho_{H}$. Energy unit is kcal/mol.

The Coulomb interaction between basic amino acid residues and acidic amino acid residues is of vital importance in the binding of the protein molecules and their biological function. As one example among many, we recently found the Coulomb interaction between the rigorously conserved arginine and glutamic acid in the membrane-embedded portion of ATP synthase plays a vital role in driving the proton-motive rotary motion [28,29]. Here, we study the Coulomb interaction between arginine (Arg) and glutamic acid (Glu) [Figure 2a]. We first calculated the potential of mean force (PMF) for the Coulomb bond formation between the Arg and Glu [Figure 2b]. We obtained the PMF as a function of the distance between the guanidyl carbon (C${}_{\zeta }$ atom) of the Arg and the carboxyl carbon (C${}_{\delta }$ atom), which was calculated by $-RT\ln[n\left(d\right)/N_{\mathrm{ref}}]$, where *R* is the gas constant, *T* is the temperature (300 K), n(d) is the number of the snapshots that fall in the range of d±0.05 Å, and $N_{\mathrm{ref}}$ is the number of the snapshots in the reference state (d=10 Å). In Figure 2b, we can see the minimum PMF at d≈4 Å both for the GB model and the explicit water model, indicating the thermodynamic stability of the Coulomb bond. It is also clearly seen that the Coulomb bond stability in the GB model increases with increasing $\rho_{H}$. We then examined the Coulomb energies in detail. In Figure 2c, we show the *total* Coulomb energy as a function of *d*, which was calculated by using the snapshot structures obtained from the MD simulations with the explicit water model (a total of 40,000 snapshot structures sampled at a 0.1 ns interval). The stability of the Coulomb bond is increased with increasing $\rho_{H}$, which is essentially the same as what was observed in the two-atom (H–O) system described above. In addition, by comparing the PMF [Figure 2b] with the total Coulomb energy [Figure 2c], we can find that the stability enhancement in the PMF is almost the same amount as that in the total Coulomb energy; the amount of the stability enhancement at d≈4 Å from $\rho_{H}$ = 1.1 Å (red) to 1.5 Å (blue) is about 4 kcal/mol for both the PMF and the total Coulomb energy. We then scrutinized the self- (desolvation) energy term (Equation (16)) and the interaction energy term (Equation (17)). As seen in Figure 2d,e, the desolvation energy increases as *d* is decreased, reflecting the desolvation penalty upon Coulomb bond formation, and the amount of the increase in $\Delta G^{\mathrm{self}}$ is enhanced with increasing $\rho_{H}$. On the other hand, the interaction energy decreases as *d* is decreased, bringing about the stability of the Coulomb bond, and the amount of the decrease in $\Delta G^{\mathrm{int}}$ is enhanced with increasing $\rho_{H}$. The $\rho_{H}$-enhanced desolvation penalty is counterbalanced by the $\rho_{H}$-enhanced Coulomb bond stability, as is clearly seen in Figure 2f,g, where the average values averaged in the Coulomb bond formation state (3.9≤d≤4.0 Å) are shown as a function of $\rho_{H}$. Here, the $\rho_{H}$-enhancement in $G^{\mathrm{int}}$ is larger than that in $\Delta G^{\mathrm{self}}$, so the former surpasses the latter, and, consequently, the Coulomb bond stability is enhanced as $\rho_{H}$ is increased. The physical reason for the weaker $\rho_{H}$-enhancement in $\Delta G^{\mathrm{self}}$ is essentially the same as explained in the two-atom system (the counterbalance between the increase in $\Delta G_{O}^{\mathrm{self}}$ and the decrease in $\Delta G_{H}^{\mathrm{self}}$ with increasing $\rho_{H}$), and, hence, the increased Coulomb bond stability with increasing $\rho_{H}$ is also observed in other basic residue–acidic residue pairs, such as the Lys–Asp pair (see Supplementary Figure S1).

### 2.3. Two-Protein System (Bn-Bs System)

**Figure 3:**
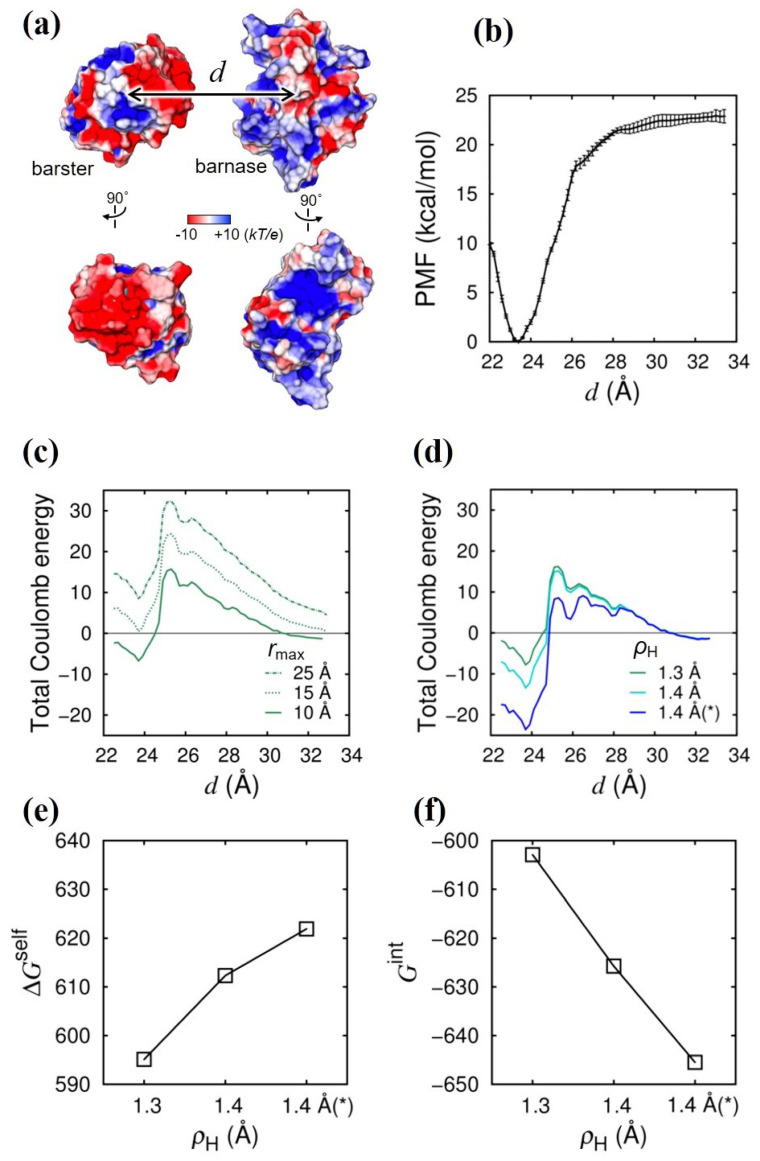
Effect of $r_{\max}$ and $\rho_{H}$ on Coulomb energies in Bn–Bs system. (**a**) Illustration of the system. Coulomb potential at the surface of Bn and Bs is shown by the color map [red: −10kT/e; blue +10kT/e, where *e* is the elementary charge, *k* is the Boltzmann constant, and *T* is temperature (300 K)]. Binding interfaces of Bn and Bs (rotated by $90^{\circ }$) are displayed beneath. Coulomb potential was calculated by APBS [34]. (**b**) Potential of mean force (PMF) is shown as a function of the intermolecular distance *d* [see (**a**)] obtained by conducting an umbrella sampling with the explicit water. (**c**) Total Coulomb energy is shown as a function of *d* at different $r_{\max}$ values ($r_{\max}=$ 25, 15, and 10 Å). (**d**) Total Coulomb energy is shown as a function of *d* at different $\rho_{H}$ values ($\rho_{H}=$ 1.3 and 1.4 Å). The asterisk (*) indicates that the intrinsic radius of the carboxyl oxygen ($\rho_{O}$) in the side chain of acidic residues is also changed (from 1.5 to 1.6 Å). (**e**) Total desolvation energy ($\Delta G^{\mathrm{self}}$) and (**f**) total interaction energy ($G^{\mathrm{int}}$), averaged in the range of 23.6≤d≤23.8 Å, are shown as a function of $\rho_{H}$. Energy unit is kcal/mol.

The binding between the bacterial ribonuclease barnase (Bn) and its inhibitor barstar (Bs) has been intensively studied both experimentally [30] and theoretically [1,2,31,32,33] because of their strong binding affinities and fast binding rates, which are largely due to the Coulomb interaction between Bn and Bs. In fact, a clear charge complementarity is seen at the binding interface [Figure 3a], where the surface of Bn is positively charged and the surface of Bs is negatively charged. We first calculated the potential of mean force (PMF) for the binding between Bn and Bs as a function of the distance between the center of mass of Bn and that of Bs, denoted by *d* [Figure 3b]. The minimum PMF is found at d=23.7 Å, which corresponds to the bound state and should be stabilized by the Coulomb interaction. Note that the non-Coulomb interaction should also contribute to the stability of the bound state in the case of the Bn–Bs binding, in which the buried surface area upon binding is much larger than that in the case of the Arg–Glu system. Accurate evaluation of the contribution of the non-Coulomb energy to the binding stability, which is needed to obtain the total binding free energy in addition to the Coulomb energy, is another important issue [23]. Here, we focus on the contribution of the Coulomb energy (we will address the contribution of the non-Coulomb energy in the next study). In contrast to the case of the Arg–Glu system, in which the water-excluded volume for an amino acid monomer is small, and, hence, the effect of the upper limit (cutoff) of the spatial integral in Equation (11), $r_{\max}$, is negligible, the water-excluded volume of a protein molecule is much larger so that the effect of $r_{\max}$ on the Coulomb energy should become much greater in the Bn–Bs system. In Figure 3c, we show the total Coulomb energy as a function of *d* at three different values of $r_{\max}$ (10, 15, and 25 Å). We can see that a large energy barrier arises when $r_{\max}$ is large, which is caused by the overestimation of the desolvation penalty in the GB model, in which the so-called Coulomb field approximation is used [24]. The overestimation of the desolvation penalty can be corrected by more elaborate, yet computationally demanding, GB models (e.g., the one developed by Onufriev and coworkers [22]); alternatively, it can also be remedied to a large extent by using a smaller $r_{\max}$ [24] in the plain and the computationally less demanding GB model [16] that we used here. However, even with $r_{\max}=10$ Å, the energy barrier remains, and the stabilization of the bound state is only a little. Therefore, keeping $r_{\max}$ at the small value of 10 Å, we studied the effect of the lower limit of the spatial integral, i.e., the intrinsic radius ρ, on the total Coulomb energy [Figure 3d]. As was the case in the H–O system and the Arg–Glu system, increasing $\rho_{H}$ (the intrinsic radius of the hydrogen atoms covalently bonded to a nitrogen atom in the side chain of Arg or Lys) has the effect of stabilizing the binding between Bn and Bs. The stabilizing effect is short ranged and localized in the close contact region with d≲25 Å. Furthermore, Figure 3e,f clearly show that the physical mechanism of the enhancement of the stabilization with increasing $\rho_{H}$ is the same as that in the H–O system and in the Arg–Glu system, i.e., the decrease in $\Delta G^{\mathrm{int}}$ (the enhancement of the Coulomb attraction) surpasses the increase in $\Delta G^{\mathrm{self}}$ (Coulomb desolvation penalty) with increasing $\rho_{H}$. The same physical mechanism of the stability enhancement of the Coulomb bonds holds true for the intrinsic radius of the carboxyl oxygen atoms in the side chain of Glu or Asp, so that Bn–Bs binding was further stabilized by increasing $\rho_{O}$ from 1.5 Å (the Bondi radius of the oxygen atom [25]) to 1.6 Å [Figure 3d–f].

## 3. Methods

### 3.1. Intrinsic Radius in GB Model

We first explain where the intrinsic radius (also referred to as the GB atomic radius) appears in the basic framework of the GB model [4].

Suppose a system composed of two spherical particles solvated in water with the effective dielectric constant ε; the two particles possess the charges $q_{1}$ and $q_{2}$ at their centers, the sphere radii are $\rho_{1}$ and $\rho_{2}$, respectively, and the distance between the centers of the particles is *d*. When we take the infinite separation of the two particles in a vacuum as the reference state, the total Coulomb energy of the above system in water at the separation distance of *d*, $G_{C}\left(d\right)$ is represented by the sum of the Coulomb (Born) solvation energy $G_{\mathrm{sol}}^{\mathrm{self}}$ at the infinite separation and the Coulomb interaction energy in water $G_{\mathrm{wat}}^{\mathrm{int}}$, i.e.,

(1) $$\begin{array}{cc} \; & G_{C}\left(d\right)=G_{\mathrm{sol}}^{\mathrm{self}}\left(\infty \right)+G_{\mathrm{wat}}^{\mathrm{int}}\left(d\right), \end{array}$$

where $G_{\mathrm{sol}}^{\mathrm{self}}$ and $G_{\mathrm{wat}}^{\mathrm{int}}$ are given by

(2) $$\begin{array}{c} G_{\mathrm{sol}}^{\mathrm{self}}=\left(\frac{1}{\varepsilon_{w}}-1\right)\frac{q_{1}^{2}}{2\rho_{1}}+\left(\frac{1}{\varepsilon_{w}}-1\right)\frac{q_{2}^{2}}{2\rho_{2}}, \end{array}$$

(3) $$\begin{array}{cc} \; & G_{\mathrm{wat}}^{\mathrm{int}}\left(d\right)=\frac{q_{1}q_{2}}{\varepsilon d}. \end{array}$$

The effective dielectric constant, ε, in Equation (3) should take the dielectric constant of bulk water ($\varepsilon_{w}$) when *d* is large enough, and it should be lowered as *d* becomes smaller because the water-excluded regions come closer. Here, if we consider solvating the system at the separation distance *d* (instead of solvating the system at infinite separation, as assumed in Equation (1)), then Equation (1) can be replaced by

(4) $$\begin{array}{cc} \; & G_{C}\left(d\right)=\left(G_{\mathrm{sol}}^{\mathrm{self}}\left(d\right)+G_{\mathrm{sol}}^{\mathrm{int}}\left(d\right)\right)+E_{\mathrm{vac}}^{\mathrm{int}}\left(d\right), \end{array}$$

where $G_{\mathrm{sol}}^{\mathrm{self}}$, $G_{\mathrm{sol}}^{\mathrm{int}}$, and $E_{\mathrm{vac}}^{\mathrm{int}}$ are given by

(5) $$\begin{array}{c} G_{\mathrm{sol}}^{\mathrm{self}}\left(d\right)=\left(\frac{1}{\varepsilon (d)}-1\right)\frac{q_{1}^{2}}{2\rho_{1}}+\left(\frac{1}{\varepsilon (d)}-1\right)\frac{q_{2}^{2}}{2\rho_{2}}, \end{array}$$

(6) $$\begin{array}{cc} \; & G_{\mathrm{sol}}^{\mathrm{int}}\left(d\right)=\left(\frac{1}{\varepsilon (d)}-1\right)\frac{q_{1}q_{2}}{d}, \end{array}$$

(7) $$\begin{array}{cc} \; & E_{\mathrm{vac}}^{\mathrm{int}}\left(d\right)=\frac{q_{1}q_{2}}{d}. \end{array}$$

The sum of $G_{\mathrm{sol}}^{\mathrm{self}}$ and $G_{\mathrm{sol}}^{\mathrm{int}}$ is called the generalized Born (GB) solvation energy [4,35], which we designate as $G_{\mathrm{gb}}$. The separation-distance dependent change in the effective dielectric constant of water, as mentioned above, is taken into consideration in the GB model proposed by Still et al. [4], where $G_{\mathrm{sol}}^{\mathrm{self}}$ and $G_{\mathrm{sol}}^{\mathrm{int}}$ are approximated by

(8) $$\begin{array}{cc} G_{\mathrm{sol}}^{\mathrm{self}}\left(d\right) & \approx G_{\mathrm{gb}}^{\mathrm{self}}\left(d\right)\\ \; & =\left(\frac{1}{\varepsilon_{w}}-1\right)\frac{q_{1}^{2}}{2R_{1}}+\left(\frac{1}{\varepsilon_{w}}-1\right)\frac{q_{2}^{2}}{2R_{2}}, \end{array}$$

(9) $$\begin{array}{cc} G_{\mathrm{sol}}^{\mathrm{int}}\left(d\right) & \approx G_{\mathrm{gb}}^{\mathrm{int}}\left(d\right)\\ \; & =\left(\frac{1}{\varepsilon_{w}}-1\right)\frac{q_{1}q_{2}}{{\left\{d^{2}+R_{1}R_{2}\exp\left[-d^{2}/\left(4R_{1}R_{2}\right)\right]\right\}}^{\frac{1}{2}}}, \end{array}$$

where $R_{i}\;\left(i=1,2\right)$ is called the effective Born radius and represents the *d*-dependent behavior of the effective dielectric constant ε around the particle *i* in such a manner that

(10) $$\begin{array}{ccc} & & \frac{1}{R_{i}}=\frac{1}{\rho_{i}}-I, \end{array}$$

(11) $$\begin{array}{ccc} & & I=\frac{1}{4\pi }\int_{\begin{array}{c} \rho_{i}\leq \left|r_{i}\right|\leq r_{\max},\\ r_{i}\in V_{\mathrm{excl}} \end{array}}\frac{1}{|r_{i}{|}^{4}}dr_{i}. \end{array}$$

The right-hand side of Equation (10) originates from the spatial integral of the energy density of the electric field due to the particle *i* [10]. The variable of the integration in Equation (11), $r_{i}$, represents the position vector, with the center of the particle *i* set at the origin; $r_{\max}$ is the cutoff distance (the upper limit) of the integral; and $V_{\mathrm{excl}}$ denotes the water-excluded volume due to the other particle over which the spatial integral runs (in fact, the integration is replaced by the summation of analytical pairwise functions [5,6]). The intrinsic radius, $\rho_{i}$, on which we focus in this study, appears here as the lower limit for the spatial integral of Equation (11). The quantity *I* acts as the measure of the surrounding dielectric environment of the particle *i*, changing from 0 for the completely solvated state to $1/\rho_{i}$ for the completely desolvated state. Accordingly, $R_{i}$ increases from $\rho_{i}$ to infinity as the desolvation proceeds (i.e., as the effective dielectric constant ε around the particle *i* decreases from $\varepsilon_{w}$ to one). Using Equations (8) and (9), $G_{\mathrm{gb}}$ can be written in a unified form as

(12) $$\begin{array}{cc} G_{\mathrm{gb}}\left(d\right) & =G_{\mathrm{gb}}^{\mathrm{self}}\left(d\right)+G_{\mathrm{gb}}^{\mathrm{int}}\left(d\right)\\ \; & =\frac{1}{2}\left(\frac{1}{\varepsilon_{w}}-1\right)\sum_{i,j}^{2}\frac{q_{i}q_{j}}{{\left\{d^{2}+R_{i}R_{j}\exp\left[-d^{2}/\left(4R_{i}R_{j}\right)\right]\right\}}^{\frac{1}{2}}}. \end{array}$$

### 3.2. Energy Analysis

We employed the following three differently sized systems in the energy analysis: (i) a two-atom system (H–O system), (ii) a two amino acid system (Arg–Glu system), and (iii) a two protein system (barstar–barnase system).

(i) Two-atom system (H–O system): The oppositely charged two-atom system [see Figure 1a], to which Equations (7)–(12) can be directly applied, was studied as the simplest system. Here, by using the charge and the intrinsic radius parameters in AMBER ff99SB [36,37] for the O${}_{\epsilon }$ atom of glutamic acid [$q_{O}=-0.82\;e$ (*e* is the elementary charge) and $\rho_{O}=1.5$ Å] and those for the H${}_{\eta }$ atom of arginine ($q_{H}=+0.45\;e$ and $\rho_{H}=1.2$ Å), we calculated the energy components in Equations (7)–(11) to obtain the Coulomb self-energy and interaction energy. To study the energy change accompanying the Coulomb bond (ionic bond) formation, we first calculated the Coulomb *self*-energy at a separation distance of *d* relative to that at infinite separation in water, i.e., the “desolvation energy”, for each atom (H or O), defined by

(13) $$\begin{array}{cc} \; & \Delta G_{X}^{\mathrm{self}}\left(d\right)=G_{\mathrm{gb},X}^{\mathrm{self}}\left(d\right)-G_{\mathrm{gb},X}^{\mathrm{self}}\left(\infty \right)\;\left(X=O,H\right), \end{array}$$

(14) $$\begin{array}{cc} \; & G_{\mathrm{gb},X}^{\mathrm{self}}\left(d\right)=\left(\frac{1}{\varepsilon_{w}}-1\right)\frac{q_{X}^{2}}{2R_{X}}, \end{array}$$

where $R_{X}$ depends on *d*, and $R_{X}=\rho_{X}$ at d=∞. We also studied the change in the *interaction* energy accompanying the Coulomb bond formation (i.e., the sum of the second and third terms of Equation (4)),

(15) $$\begin{array}{cc} G^{\mathrm{int}}\left(d\right) & =E_{\mathrm{vac}}^{\mathrm{int}}\left(d\right)+G_{\mathrm{sol}}^{\mathrm{int}}\left(d\right)\\ \; & \approx E_{\mathrm{vac}}^{\mathrm{int}}\left(d\right)+G_{\mathrm{gb}}^{\mathrm{int}}\left(d\right)\\ \; & =\frac{q_{H}q_{O}}{d}+\left(\frac{1}{\varepsilon_{w}}-1\right)\frac{q_{H}q_{O}}{{\left\{d^{2}+R_{H}R_{O}\exp\left[-d^{2}/\left(4R_{H}R_{O}\right)\right]\right\}}^{\frac{1}{2}}}. \end{array}$$

Then, we calculated $\Delta G_{X}^{\mathrm{self}}\left(d\right)$ and $G^{\mathrm{int}}\left(d\right)$ by systematically changing $\rho_{H}$. The $r_{\max}$ in Equation (11) was set to 999 Å (or effectively no cutoff for the spatial integral). The bulk dielectric constant of water, $\varepsilon_{w}$, was set to 78.5 throughout this study.

(ii) Two amino acid system (Arg–Glu system): As an example of the Coulomb bond (salt bridge) formation between amino acid residues, we studied the interaction between the side chain of arginine (Arg) and that of glutamic acid (Glu) [see Figure 2a]. The main-chain amino terminal and the carboxyl terminal of the Arg and Glu were neutralized by acetylation and methylamidation, respectively, and the Arg and Glu were aligned so that the main-chain orientation (the vector connecting the methyl carbons in the terminal acetyl and methylamide groups) of the Arg becomes parallel to that of the Glu, and the side-chain orientation of the Arg (the vector connecting the C${}_{\alpha }$ and C${}_{\beta }$ atoms) faces that of the Glu. The distance between the C${}_{\alpha }$ atoms of the Arg and Glu was set to 14 Å. After energy minimization and subsequent equilibration at 300 K, in which the main-chain heavy atoms were positionally restrained with a spring constant of 1000 kcal/mol/Å${}^{2}$ to relax the side-chain conformation, four independent 1-μs MD runs were conducted at 300 K, with the main-chain heavy atoms positionally restrained by a spring constant of 20 kcal/mol/Å${}^{2}$. The temperature was controlled using the Langevin thermostat with a collision frequency of 1.0 ps${}^{-1}$. Throughout the present study, we employed the GB model refined by Onufriev et al. [16], using the GB${}^{\mathrm{OBC}}$(II) parameter set. In this study, the intrinsic radius of the hydrogen atom covalently bonded to a nitrogen atom, denoted by $\rho_{H}$, was systematically changed in the range from 1.1 to 1.5 Å, in which the default value (the mBondi2 value) of 1.3 Å [16] is included.

For the purpose of comparison, we used the explicit water model for the MD simulation, in addition to the GB solvation model. We immersed the Arg and Glu in a water box containing 2337 water molecules (the TIP3P water model [38] was used), to which the periodic boundary condition and the particle mesh Ewald method [39] (with a direct space cutoff of 8 Å) were applied. After energy minimization and subsequent equilibration at 300 K and 0.1 MPa (using the Berendsen barostat [40]), in which the main-chain heavy atoms were positionally restrained with a spring constant of 1000 kcal/mol/Å${}^{2}$ to relax the side-chain conformation and the system volume, four independent 1-μs MD runs were conducted at 300 K under a constant volume condition, with the main-chain heavy atoms positionally restrained by a spring constant of 20 kcal/mol/Å${}^{2}$. We employed AMBER [41] (version 12 or later) with the ff99SB force field [36,37] for both MD simulations with the GB solvation model and the explicit solvent model. For each solvation model, we obtained the potential of mean force (PMF) as a function of the distance between the C${}_{\zeta }$ atom (guanidyl carbon) of the Arg and the C${}_{\delta }$ atom (carboxyl carbon) of the Glu to measure the stability of the Coulomb bond between the Arg and Glu.

For each of the snapshot structures obtained from the MD runs conducted in explicit water (a total of 40,000 snapshot structures sampled at a 0.1 ns interval), we calculated the total *self*- (or desolvation) energy and the total *interaction* energy, defined by

(16) $$\begin{array}{cc} \Delta G_{}^{\mathrm{self}}\left(d\right) & =\sum_{i}^{N}\left[G_{\mathrm{gb},i}^{\mathrm{self}}\left(d\right)-G_{\mathrm{gb},i}^{\mathrm{self}}\left(\infty \right)\right], \end{array}$$

(17) $$\begin{array}{cc} G_{}^{\mathrm{int}}\left(d\right) & =\sum_{k<l}\left[E_{\mathrm{vac},kl}^{\mathrm{int}}\left(d\right)+G_{\mathrm{gb},kl}^{\mathrm{int}}\left(d\right)\right], \end{array}$$

where $G_{\mathrm{gb},i}^{\mathrm{self}}$ conforms to Equation (14), *N* is the total number of the atoms in the Arg and Glu (N=63), and $E_{\mathrm{vac},kl}^{\mathrm{int}}$ and $G_{\mathrm{gb},kl}^{\mathrm{int}}$ conform to Equation (15) (*k* and *l* are the atom identifiers that belong to the Arg and Glu, respectively). Then, we calculated $\Delta G_{}^{\mathrm{self}}\left(d\right)$ and $G^{\mathrm{int}}\left(d\right)$ by systematically changing $\rho_{H}$. The $r_{\max}$ was set to 999 Å (or effectively no cutoff for the spatial integral).

(iii) Two-protein system (barstar–barnase system): As an example of the Coulomb bond (salt bridge) formation between protein molecules, we studied the binding between barnase (Bn) and barstar (Bs) [see Figure 3a]. We solvated the Bn–Bs complex (PDB ID: 1BRS) [30] in a water box containing 15,611 water molecules (the TIP3P [38] model was used) and four sodium ions [42] to neutralize the system, and we employed the AMBER ff03 force field [43]. We set all of the basic and acidic residues in the charged state and all histidines in the single-protonation state (ϵ-protonated state). The missing residues (N-terminal two residues in Bn) were complemented by Modeller [44]. We then conducted the umbrella sampling in explicit water to obtain the potential of mean force of the Bn–Bs binding (as a function of the separation distance between the center of mass of the Bn and that of the Bs, denoted by *d*). We conducted the MD simulation using AMBER [41] (version 12 or later) at a constant temperature (set to 300 K using the Langevin thermostat with a collision frequency of 1.0 ps${}^{-1}$) and at a constant volume (the system volume was relaxed in the preparation run conducted at 0.1 MPa using the Berendsen barostat [40]).

For the umbrella sampling, we applied the umbrella potential, $\frac{1}{2}a_{i}{\left(d-d_{i}\right)}^{2}$ (i=1,2,...,19) for the *i*-th window and changed $d_{i}$ from 22 to 32 Å at an interval of 0.5 Å for $d_{i}\leq 28$ with $a_{i}=9.6$ kcal/mol/Å${}^{2}$ (two windows were added at $d_{i}=25.75$ and 26.75 Å to improve the overlap between the distributions of the neighboring windows). For $d_{i}>28$ Å, we changed $d_{i}$ at an interval of 1 Å with $a_{i}=2.4$ kcal/mol/Å${}^{2}$. During the umbrella sampling, to maintain the relative orientation between the Bn and Bs and the side-chain conformations of the residue involved in the binding interface, we employed the same restraints as those in the study by Gumbart et al [33]. For each of the 19 windows, we conducted five independent 5 ns MD runs and used the weighted histogram analysis method (WHAM) [45] to obtain the PMF as a function of *d*.

Using the snapshot structures obtained from the umbrella sampling [snapshots sampled at a 0.1 ns interval from the last 1 ns of the five 5 ns MD runs (i.e., total 50 snapshots) in each window, we calculated the total desolvation energy, $\Delta G_{}^{\mathrm{self}}\left(d\right)$, and the total interaction energy, $G^{\mathrm{int}}\left(d\right)$, in the same way as in Equations (16) and (17). Then, we studied the effect of $\rho_{H}$ (the intrinsic radius of the hydrogen atom covalently bonded to a nitrogen atom in the side chain of Arg or Lys) on $\Delta G_{}^{\mathrm{self}}$ and $G^{\mathrm{int}}$. For the Bn–Bs system, we also studied the effect of the upper limit of the spatial integral, $r_{\max}$, on $\Delta G_{}^{\mathrm{self}}$ and $G^{\mathrm{int}}$ by using different values for $r_{\max}$ (10, 15, and 25 Å).

## 4. Concluding Remarks

We clarified the physical mechanism of how the Coulomb bond stability is increased as the intrinsic radius, ρ, is increased in the generalized Born (GB) model. As ρ is increased, the desolvation penalty upon Coulomb bond formation is increased. However, due both to the counterbalance between the desolvation energies of the two interacting atoms and to the enhancement of the Coulomb interaction as a result of the enlarged region with a low effective dielectric constant, the desolvation penalty is more than compensated for by the enhanced stabilization of the Coulomb interaction energy. We note that increasing the intrinsic radius, the effect of which is the alleviation of the desolvation penalty and the enhancement of the attractive interaction, can be regarded as an effective modeling of the intercalating water molecules at the binding interface, which have a crucial role in strengthening the binding affinity [46]. Practically, to study the binding between proteins using the GB model (with the Coulomb field approximation), which is fast yet tends to overestimate the Coulomb desolvation energy, it is worthwhile to consider adjusting the two parameters in combination, i.e., the lower and the upper limits for the spatial integral of the energy density of the electric field around a charged atom, ρ and $r_{\max}$, respectively. The use of a larger ρ for the Coulomb-bond-forming hydrogen and oxygen atoms strengthens the Coulomb attraction in the short range. The use of a relatively small $r_{\max}$ alleviates the overestimated desolvation penalty in the long range. In the case of the binding between barnase and barstar, the above-mentioned two-way adjustment was found to work well for the intermolecular binding driven by the Coulomb attraction. Investigating whether the same two-way strategy can be applied to other systems, as well as the investigation of the contribution of the non-Coulomb energy to the binding, remains for a future work.

## Data Availability

The data presented in this study are available in Supplementary Material.

## Supplementary Materials

The following supporting information can be downloaded at: https://www.mdpi.com/article/10.3390/ijms24054700/s1.
