# Supplementary material for: The Intrinsic Radius as a Key Parameter in the Generalized Born Model to Adjust Protein-Protein Electrostatic Interaction
Source: Int J Mol Sci. 2023 Feb 28;24(5):4700. doi: 10.3390/ijms24054700 (PMC10003186; doi:10.3390/ijms24054700)
Supplement: Supplementary file 1 [file ijms-24-04700-s001.zip › ijms-2179156-supplementary.pdf]

The intrinsic radius as a key parameter in the generalized Born model to adjust protein-protein electrostatic interaction (Supplementary Figure)

Dan Parkin[1] and Mitsunori Takano*[2]

[1] *Research Institute for Science and Engineering, Waseda University, Ohkubo 3-4-1, Shinjuku-Ku, Tokyo 169-8555, Japan*

[2] *Department of Pure and Applied Physics, Research Institute for Science and Engineering, Waseda University, Ohkubo 3-4-1, Shinjuku-Ku, Tokyo 169-8555, Japan*

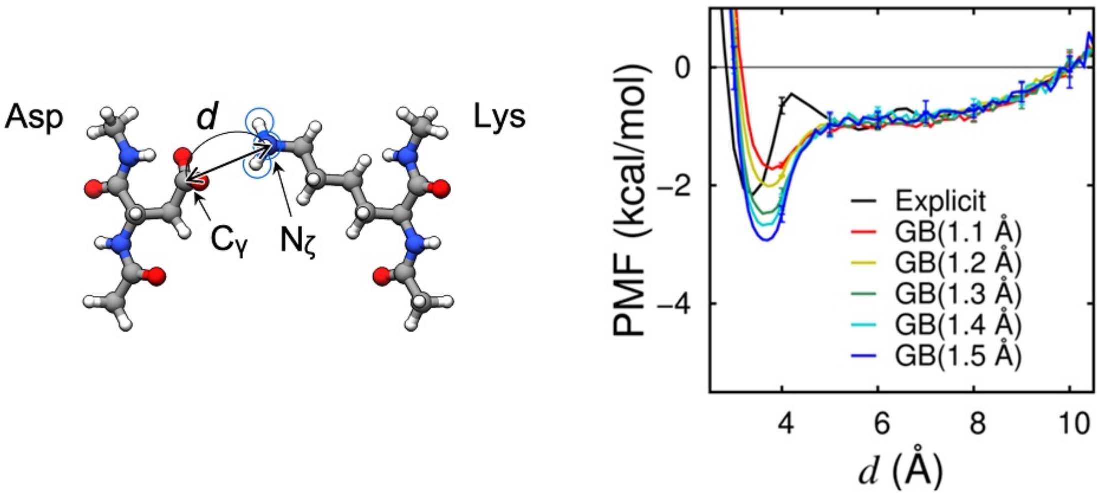

FIG. S1. Effect of $\rho_{\rm H}$ on Coulomb bond formation in Lys-Asp system. (Left) Illustration of the system. The intrinsic radii of the hydrogen atoms in the ammonium group of Lys (encircled), denoted by $\rho_{\rm H}$, were uniformly and systematically changed from 1.1 Å to 1.5 Å. (Right) Potential of mean force (PMF) is shown as a function of d (see left) for the explicit water model (black) and for the GB solvation model at different $\rho_{\rm H}$ values (1.1 to 1.5 Å). The Lys-Asp system was prepared in the same manner as the Arg-Glu system, with the distance between C_α atoms of Lys and Asp set at 11 Å.
